# Pan-genome assembly of vine tea (*Nekemias grossedentata*) reveals structural variation in its dihydromyricetin biosynthesis diversity

**DOI:** 10.1093/hr/uhaf307

**Published:** 2025-11-18

**Authors:** Danlu Han, Songyan Na, Zhuangwei Hou, Fangping Li, Chengluo Zhu, Yingying Li, Yingzi Zheng, Qiong Mo, Jiaqi Chen, Simin Xia, Xiaofan Zhou, Chengwei Yang, Jun Liu

**Affiliations:** Guangdong Provincial Key Laboratory of Biotechnology for Plant Development, School of Life Science, South China Normal University, Guangzhou 510631, China; Guangdong Provincial Key Laboratory of Biotechnology for Plant Development, School of Life Science, South China Normal University, Guangzhou 510631, China; Shenzhen Branch, Guangdong Laboratory of Lingnan Modern Agriculture, Genome Analysis Laboratory of the Ministry of Agriculture and Rural Affairs, Agricultural Genomics Institute at Shenzhen, Chinese Academy of Agricultural Sciences, Shenzhen 518120, China; Guangdong Provincial Key Laboratory of Plant Molecular Breeding, State Key Laboratory for Conservation and Utilization of Subtropical Agro-Bioresources, South China Agricultural University, Guangzhou 510642, China; Guangdong Provincial Key Laboratory of Biotechnology for Plant Development, School of Life Science, South China Normal University, Guangzhou 510631, China; Guangdong Provincial Key Laboratory of Biotechnology for Plant Development, School of Life Science, South China Normal University, Guangzhou 510631, China; Guangdong Provincial Key Laboratory of Biotechnology for Plant Development, School of Life Science, South China Normal University, Guangzhou 510631, China; Guangdong Provincial Key Laboratory of Biotechnology for Plant Development, School of Life Science, South China Normal University, Guangzhou 510631, China; Guangdong Provincial Key Laboratory of Biotechnology for Plant Development, School of Life Science, South China Normal University, Guangzhou 510631, China; Guangdong Provincial Key Laboratory of Biotechnology for Plant Development, School of Life Science, South China Normal University, Guangzhou 510631, China; Guangdong Provincial Key Laboratory of Plant Molecular Breeding, State Key Laboratory for Conservation and Utilization of Subtropical Agro-Bioresources, South China Agricultural University, Guangzhou 510642, China; Guangdong Provincial Key Laboratory of Biotechnology for Plant Development, School of Life Science, South China Normal University, Guangzhou 510631, China; Guangdong Key Laboratory for Crop Germplasm Resources Preservation and Utilization, Agro-Biological Gene Research Center, Guangdong Academy of Agriculture Sciences, Guangzhou 510640, China

## Abstract

Vine tea (*Nekemias grossedentata*) is a dual-purpose medicinal and edible liana with a documented history of consumption in China spanning millennia. It has been extensively utilized among ethnic minority groups, including the Tujia, Yao, and Dong communities, for at least 700–1000 years, where it is traditionally revered as the ‘Immortal Herb’ or ‘Longevity Tea’. This study reports the haplotype-resolved chromosome-scale genomes of two major cultivated diploid vine tea accessions (*N. grossedentata*, 2*n* = 40). Phylogenetic analysis revealed that *N. grossedentata* diverged from Cissus rotundifolia ~26.27 million years ago (MYA) and from *Vitis vinifera* around 17.30 MYA. Comparative genomic analysis within the genus uncovered species-specific evolutionary patterns. Furthermore, we constructed a pan-genome encompassing 39 vine tea cultivars and characterized structural variations among cultivated varieties. Correlation analysis between dihydromyricetin (DMY) content and leaf transcriptomes across these cultivars identified ~1 kb presence/absence variations (PAVs) associated with the expression of *F3′5′H*, a gene critical for DMY biosynthesis in vine tea. Collectively, this genomic resource provides a valuable foundation for advancing herbal crop breeding and development, while offering insights into the biosynthetic pathways underlying specialized metabolism in Vitaceae.

## Introduction

Vine tea (*Nekemias* spp.), also known as ‘moyeam’, is a perennial woody liana belonging to the Vitaceae family. It is primarily cultivated in warm and humid environments, such as forests, mountain slopes, and shrublands [1]. The plant is characterized by its distinctive ‘white frost’ appearance and a unique flavor profile—initially slightly bitter with a lingering sweet aftertaste. Due to these sensory qualities, vine tea is commonly brewed and consumed in a manner similar to green tea. The earliest documented use of vine tea dates back to the Yuan Dynasty (1330s), when the medical text Yinshan Zhengyao (
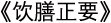
) recorded its consumption and medicinal properties [2], marking a history of at least 694 years. In recent years, scientific investigations have revealed that vine tea possesses a range of significant bioactive properties, including antioxidant, anti-inflammatory, and neuroprotective effects [3]. These attributes position it as a potential natural antioxidant, leading to its widespread application in nutraceuticals, pharmaceuticals, and cosmetic formulations.

Vine tea is an edible plant with important medicinal values. Its young stems, tendrils, and leaves are rich in amino acids, vitamins, flavonoids, glycosides, phenolic substances, and a variety of essential trace elements. According to the folk long-term drinking confirmed that vine tea has the efficacy of thirst quenching, sweet throat, and lung, clearing heat, and detoxification, antibacterial and anti-inflammatory, and enhance immunity. Current research analyses show that vine tea extract can scavenge free radicals and block the peroxidation of lipid compounds, thus maintaining the normal function of cell membranes; it can also reduce vascular permeability and brittleness to protect blood vessels from damage. Clinical evidence suggests that vine tea extract has significant efficacy in the treatment of respiratory conditions, including upper respiratory tract infections, chronic bronchitis, and acute/chronic pharyngitis [4–7]. In addition, relevant studies have demonstrated significant modulatory effects of vine tea extracts on cardiac metabolism, antihypertensive, hypoglycaemic, and hypolipidemic aspects [8, 9], thereby suggesting potential applications in metabolic disorder therapeutics.

*Nekemias grossedentata* is particularly rich in flavonoids, with 31 distinct flavonoid compounds identified to date, accounting for ~40% of its dry weight [3, 10]. Among these, dihydromyricetin (DHM, 3,5,7,3′,4′,5′-hexahydroxy-2,3-dihydroflavonol, C_15_H_12_O_8_) is the most bioactive and extensively studied constituent [11]. DHM, a flavonoid also found in various fruits and vegetables, contains multiple reactive hydroxyl groups that interact with free radicals to form stable semiquinone radicals, thereby exhibiting potent antioxidant activity. Due to this property, DHM has been clinically applied for its antitumor, anti-inflammatory, and antimicrobial effects [12, 13]. Furthermore, its exceptional antioxidant capacity has led to widespread applications in the food and cosmetic industries [14].

In this study, we assembled chromosome-level and haplotype-resolved reference genomes for the predominant cultivated varieties of *N. grossedentata* (green-leaf and red-leaf types) in southern China. Integrated with whole-genome resequencing data from 37 cultivated accessions collected across diverse geographic regions in China, we constructed the first pan-genome for this species, thereby providing a fundamental genomic resource for investigating the genetic determinants underlying DHM content variation. Our analyses not only reconstructed the evolutionary trajectory of *N. grossedentata* within Vitaceae but also delineated the genomic basis of its specialized secondary metabolite biosynthesis pathways. Furthermore, systematic comparative genomic approaches revealed molecular signatures associated with its adaptive evolution. These findings establish a comprehensive framework for future studies on genetic diversity, molecular breeding, and functional gene discovery in *N. grossedentata*, while simultaneously advancing our understanding of speciation processes and secondary metabolic evolution in Vitaceae.

## Results

### Haplotype-resolved assembly of the main cultivars of *N. grossedentata* (LY and HY) in China

**Figure 1:**
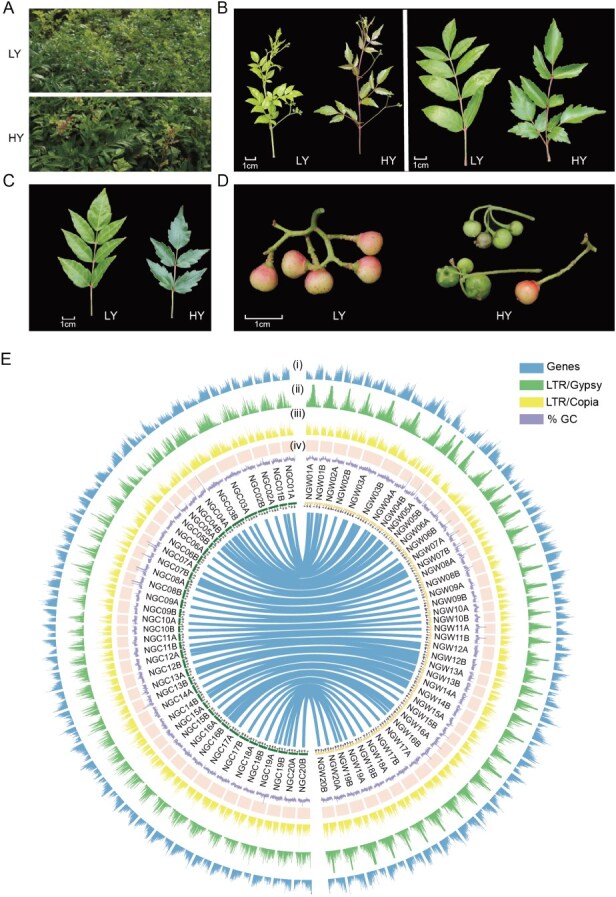
Morphological characteristics and chromosomal elements distribution of the two *Nekemia grossedentata* cultivars*.* (A–D) Photos of leaves and fruits of two species of *N. grossedentata*. LY represents the cultivar originating from Zhangjiajie, Hunan Province, HY represents the cultivar originating from Lianzhou, Guangdong Province, and Bar =1 cm. (E) Characteristics of the *N. grossedentata* assembled genome, (i) Gene density, (ii) Gypsy family of LTR density, (iii) Copia family of LTR density (iv) GC content, (v) chromosomes in the *N. grossedentata* genome, block size = 15 kb

In order to establish a comprehensive genomic resource of *N. grossedentata* (commonly known as vine tea), we carried out in-depth genome sequencing of two representative cultivars, LY (from Zhangjiajie, Hunan Province, China) has green leaves, whereas HY (from Lianzhou, Guangdong Province) bears purple leaves (Fig. 1A–D). As demonstrated in earlier studies, green leaf extracts have been shown to be the most effective in scavenging free radicals, while red leaf extracts have been found to exhibit strong antioxidant capacity in lipid systems. Both *N. grossedentata* have been identified as having significant industrial value [15]. The genome size of LY was first estimated by flow cytometry to be ~640 Mb (Fig. S1A). We then performed a genome survey using next-generation whole-genome sequencing to confirm the genome size. Analysis using the GenomeScope software showed that the genome size of LY was ~664 Mb, with a heterozygosity rate of 0.84% (Fig. S1B).

To obtain a high-quality haplotype-resolved genome assemblies, sequencing was performed using PacBio HiFi. A total of 44.58 Gb of data were obtained for LY and 39.10 Gb for HY. These were then assembled using Hifisam software after the removal of low-quality and contaminating sequences, resulting in draft assemblies of 1.25 and 1.28 Gb in size. The initial genome of LY (tagged as NGW in genome annotations) contained 1204 scaffolds and the initial genome of HY (tagged as NCW in genome annotations) contained 1294 scaffolds, with contig N50 values of 23.87 and 23.89 Mb, respectively (Table 1). Using the 3D-DNA pipeline, the primary assembly data were aligned with the Hi-C data of 71.06 Gb from LY and 73.93 Gb from HY, respectively, and finally the data from both groups were anchored to 40 pseudochromosomes (20 for each haplotype-resolved assembly). The lengths of the two haplotype genomes obtained were LY-HapA (NGW-A):599.03 Mb, LY-HapB (NGW-B): 596.56 Mb, HY-HapA (NGC-A):608.16 Mb and HY-HapB (NGC-B):607.23 Mb (Fig. 1E, Table 1 and Fig. S2). To assess genomic integrity and continuity, QV and LAI were calculated for the four haplotype-resolved assemblies. All four assemblies exhibited QV values above 41 and LAI reached more than 18 (Table 1). Strong collinearity was observed between haplotypes within each cultivar and between cultivars, indicating that our haplotype-resolved *N. grossedentata* assemblies possess high completeness and accuracy, with LY-HapB (NGW-B) designated as the primary haplotype due to its superior quality. By integrating ab initio prediction, homology evidence, and transcriptome data, a total of 30 285 to 30 507 protein-coding genes were identified in the vine tea genome. BUSCO analysis showed that the completeness of the genome reached 89.00% to 93.9% (Table 1). Functional annotations were then performed on the final data set using the eggNOG database (Fig. S3).

**Table 1:** Statistics for *A. grosedentata* ‘LY’ and ‘HY’ Haplotype-resolved genomes

	LY_HapA	LY_HapB	HY_HapA	HY_HapB
Contig N50 (bp)	23 868 265	24 874 424	25 939 893	22 446 518
scaffolds N50 (Mb)	30	30	30	30
Longest contig (bp)	48 235 221	46 653 298	34 312 929	39 010 962
Chromosome number	20	20	20	20
Number of contigs	55	73	49	54
Number of scaffolds	20	20	20	20
Genome-size (bp)	599 046 017	596 584 542	608 171 335	607 246 788
Rate of anchoring (%)	99.70	99.71	99.85	99.83
GC content (%)	34.00	34.01	34.06	34.05
BUSCO_genome (viridiplantae_odb10)	C: 98.8% [S: 97.4%, D: 1.4%], F: 0.9%, M: 0.3%, *n*: 425	C: 99.2% [S: 97.6%, D: 1.6%], F: 0.7%, M: 0.1%, *n*: 425	C: 99.2% [S: 97.6%, D: 1.6%], F: 0.7%, M: 0.1%, *n*: 425	C: 98.8% [S: 97.4%, D: 1.4%], F: 0.7%, M: 0.5%, *n*: 425
Transposable elements (%)	51.46	51.72	51.58	51.65
Merqury_out.qv	41	41	44	44
LAI	18.43	18.59	19.11	13.72
Number of predicted genes	30 403	30 314	30 507	30 285
BUSCO_protein (viridiplantae_odb10)	C: 91.5% [S: 88.2%, D: 3.3%], F: 6.1%, M: 2.4%, *n*: 425	C: 93.9% [S: 90.8%, D: 3.1%], F: 4.5%, M: 1.6%, *n*: 425	C: 90.3% [S: 72.7%, D: 17.6%], F: 8.2%, M: 1.5%, *n*: 425	C: 90.6% [S: 89.9%, D: 0.7%], F: 7.3%, M: 2.1%, *n*: 425

C: complete BUSCOs, S: complete and single-copy BUSCOs, D: complete and duplicated BUSCOs, F: fragmented BUSCOs, M: missing BUSCOs, 425: total BUSCO groups searched.

### Genetic variation between the two haplotype-resolved genomes in *N. grossedentata*

**Figure 2:**
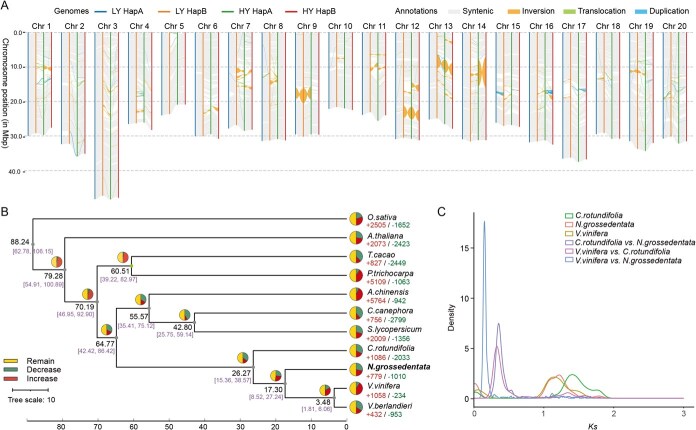
Genome evolution of *N. grossedentata.* (A) Distribution of structural variation among four haplotype-resolved genomes of the two vine tea cultivars, LY and HY. (B) The single-copy genes forming the basis of the phylogenetic tree demonstrate the number of gene families undergoing expansion and contraction, as well as the divergence time for 11 species. The pie charts illustrate the proportion of expanding, contracting, and conserved gene families among all gene families. The estimated divergence time is presented in millions of years. The tree is rooted with *O. sativa* as the outgroup. (C) The synonymous substitution rates (Ks) of *N. grossedentata*, *C. rotundifolia* and *V. vinifera*

To evaluate the genetic divergence between the two cultivars of *N. grossedentata*, we compared their respective haplotype-resolved genomes (four haplotypes in total) using the Synteny and Rearrangement Identifier (SyRI) tool [16]. There were 808 729 single nucleotide polymorphisms (SNPs), 74 966 insertions, 75 116 deletions, 6470 highly divergent regions (HDRs) and 18 duplications between the two haplotypes of LY, HapA and HapB; there were 1 616 881 SNPs, 140 901 insertions, 139 590 deletions, 14 116 HDRs, and 41 duplications between the two haplotypes of HY (Table S2). At the same time, we also found that HapA and HapB of LY had a large inversion on chromosome 9, while HapA and HapB of HY had five large inversions on chromosomes 12, 13 and 14. Compared with HapB of LY, HapA of HY had three large inversions on chromosomes 9, 12 and 13 (Fig. 2A). The regions of these groups of SVs were annotated, and GO analysis of these SV-affected genes revealed that they were mainly enriched in several biological processes, terpenoid metabolic process, and isoprenoid metabolic process (Fig. S4).

The difference in genome size between the two cultivars of vine tea varieties was 8–10 Mb. To elucidate the basis of this variation, we comparatively analyzed their gene content, repeat sequences, and gene lengths. Transposable elements (TEs) accounted for 51.46% of the HapA genome and 51.72% of the HapB genome, whereas in the HY, the TE content HapA and HapB was 51.58% and 51.82%, respectively (Table 1). Notably, although the LY HapA genome is 2.5 Mb larger than HapB, its TE content is about HapA is 5% lower (Table 1). Structural variation analysis between haplotypes revealed that the most pronounced structural difference between LY HapA and HapB occurs on chromosome 7, where ~2.25 Mb is deleted in HapB relative to HapA (Fig. S5A). This region harbors numerous tandemly duplicated genes primarily associated with the glutamate receptor signaling pathway (Fig. S5B). Comparative synteny analysis with *Vitis vinifera* and *Cissus rotundifolia* showed that this gene block is correlated with chromosome 7 of *V. vinifera* and chromosome 2 in *C. rotundifolia*, suggesting that it was deleted in LY-hapB (Fig. S5C). Furthermore, the lower TE proportion in the LY HapA was mainly attributable to a reduced number of TIR families compared with haplotypes (Table S1). The distribution of LTR-RT insertion times shows that ~0.5 MYA, LTR-RT in HapA, and HapB of the HY genome expanded rapidly and continuously (Fig. S5E).

### Comparative genomic analysis of *N. grossedentata*

In order to investigate the evolutionary history of *N. grossedentata*, a phylogenetic tree was constructed, including *Vitis vinifera*, *V. berlandieri*, *C. rotundifolia* from the Vitaceae family, *Theobroma cacao*, *Solanum lycopersicum*, *Actinidia chinensis*, *Coffea canephora*, *Populus trichocarpa*, and *Arabidopsis thaliana* from other families, with the *Oryza sativa* designated as the outgroup. The construction of the phylogenetic tree utilizing 804 single-copy orthologous genes shared by the 11 species has enabled the estimation of the time of species divergence. The divergence of *N. grossedentata*—*C. rotundifolia*, and *N. grossedentata*—*V. vinifera* was estimated to have occurred ~26.67 million years ago (MYA) and 17.30 MYA, respectively (Fig. 2B). Collinearity analysis revealed a clear 1:1 correspondence between most chromosomal regions of *N. grossedentata* and *V. vinifera* (Fig. S6), indicating that *N. grossedentata* did not undergo an independent whole-genome duplication (WGD) event after its divergence from *V. vinifera*. Meanwhile, the distribution of synonymous substitution rates (Ks) between homologous segments of homologous gene pairs in these three species was estimated, and the Ks values further confirmed this finding (Fig. 2C). We identified homologous gene families in 11 species using CAFE5, with 770 gene families expanding and 1010 contracting in the *N. grossedentata* genome. To understand the biological roles of these genes, we annotated their functions by mapping them to the Kyoto Encyclopedia of Genes and Genomes (KEGG) database. KEGG analyses showed that gene families that showed significant amplification were significantly enriched in diterpene and sesquiterpene biosynthesis and metabolism pathways, whereas contracted families were mainly enriched in the phenylpropanoid biosynthetic process and the lignin biosynthesis pathway (Fig. S7). These results suggest that terpene biosynthesis exhibits an increased lineage, which may promote the accumulation of antioxidant components in *N. grossedentata* leaves and plant environmental adaptation.

### Construction of the pan-genome of *N. grossedentata* and evolution of duplicate genomes

**Figure 3:**
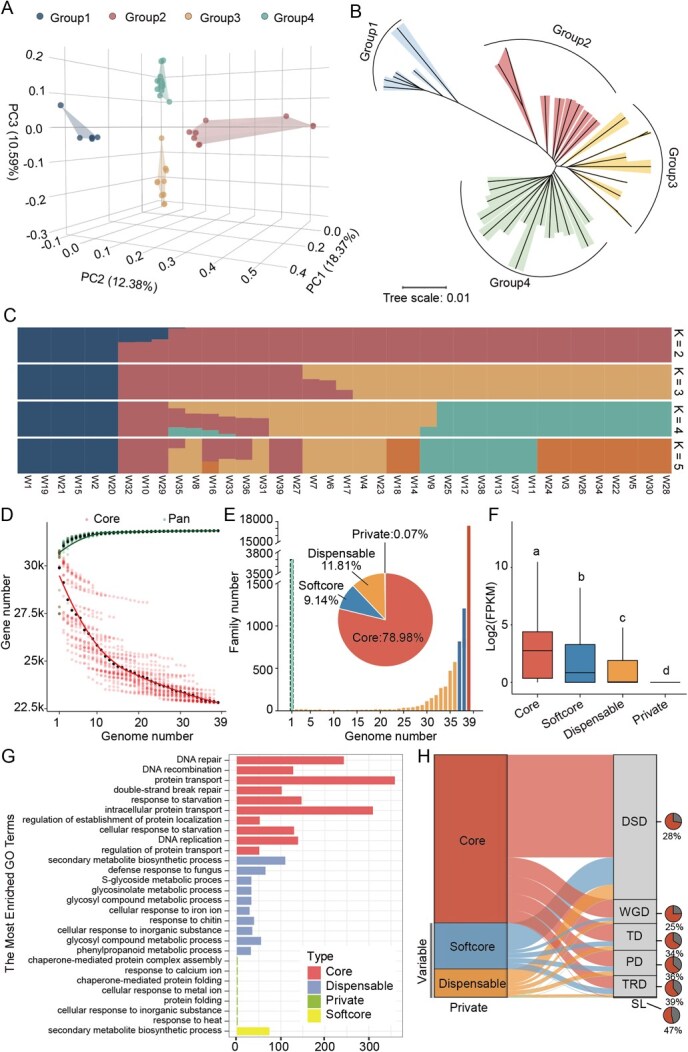
Geographic distribution and genomic characteristics of species of *N. grossedentata* in South China. (A) PCA analysis of 39 *N. grossedentata* accessions, with each accession grouped into distinct clusters; each cluster corresponds to a group consistently referenced throughout the figure. (B) Phylogenetic tree of 39 *N. grossedentata* accessions, with group assignments consistent with those shown in A. (C) Mixed analysis for *k* = 2–5. (D) The number of pan-genes and core genes in different species combinations is presented in the following table. (E) The relative proportions of pan-genes in the core, soft-core, dispensable, and private categories. The blue blocks with dashed borders represent unclustered genes and genes that are clustered only in a single genome. (F) The distribution of expression levels (in leaf tissue) of core genes, soft-core genes, dispensable genes, and private genes. The different letters above bars indicate significant differences via Duncan’s post hoc test (*P* < 0.05). (G) The GO analysis of core genes, soft-core genes, dispensable genes, and private genes. (H) The overall proportions of different types of duplicated genes (WGD, TD, PD, TRD, DSD, and single genes) in four pan-genes, where soft-core genes, dispensable genes, and private genes are all classified as variable genes.

The production of vine tea in China is primarily concentrated in the provinces of Guangdong, Guangxi, Hunan, and Yunnan, located south of the Yangtze River [3]. A total of 39 vine tea cultivars, including LY and HY, were resequenced. These materials collected by the Guangdong Academy of Agricultural Sciences (GGAS) from local producers/famer across Hunan, Guangdong, Guangxi, and Jiangxi provinces and subsequently transplanted into the germplasm nursery at GAAS for conservation. The average sequencing depth was ~40× (Table S3). Following alignment of the data with the vine tea LY hapB reference genome (selected because it represents the highest-quality assembly among available haplotypes), 60.38 million variants were identified, comprising 52.57 million single nucleotide polymorphisms (SNPs) and 7.81 million insertions/deletions (Indels) (Table S3). Following the application of filters for minor allele frequency ≥ 0.05 and missing genotype rate < 0.1, 10 139 983 high-quality SNPs were identified. These were then used to construct phylogenetic trees and perform population structure analysis. According to the population structure analysis based on genome-wide SNPs, we divided the cultivars in China into four groups, namely Group 1 (including LY), 2, 3(including HY), and 4. Group 1 is mainly distributed in the central and western parts of South China, and its color is basically dark blue, Group 2 and Group 3 included cultivated accessions from western Guangdong, and Group 4 consisted of cultivated accessions from eastern Guangdong (Fig. 3A–C). These findings indicate that the population structure of vine tea is strongly associated with its geographic distribution. Nucleotide diversity (π) for each group was estimated, which showed that the π of Group 1 germplasm was lower than that of the other three groups, and Group 2 showed higher complexity. Gene flow analysis was conducted with TreeMix, revealing that Chinese vine tea might have been domesticated in central China at first and then spread to southern regions (Fig. S8).

In order to investigate genome structure changes during *N. grossedentata* cultivation, pan-genomic analyses were performed (including chromosome-level genomes of two cultivars and 37 re-sequenced cultivars by individual un-map assembly) on 39 cultivars (Fig. 3D and E). The constructed pan-genome was annotated to 21 992 gene families (31 838 genes) and 3687 unassigned genes (Table S4). According to previously reported pan-genome articles [17], these gene families were categorized as follows: 17370 gene families for Core genes (present in all 39 accessions, containing 22 826 genes); 2010 gene families for Softcore genes (present in 37–38 accessions, containing 3409 genes), Dispensable with 2597 gene families (present in 2–36 accessions, containing 5554 genes) and Private with 15 gene families (present in only one accession, containing 49 genes). The core genes exhibit the highest expression level, indicating that they occupy a dominant position among the gene types. This is followed by the softcore and dispensable genes, while the private genes have the lowest expression level (Fig. 3F).

The annotated genes were subjected to functional enrichment and GO analysis, which revealed that the core genes were predominantly enriched in the biological process of establishment of protein localization and the pathway of intracellular protein transport. In comparison, the softcore and dispensable genes were predominantly enriched in the biological processes of secondary metabolic process and defense response to fungus, as well as the pathway of glycosyl compound metabolic process. The private genes mainly function in response to calcium ion and response environment stress (Fig. 3G). In addition, we also detected the duplication types of different genes. The core genes mainly underwent dispersed duplicates (DSD) and whole-genome duplication (WGD), softcore and dispensable genes underwent tandem duplicates (TD), proximal duplicates (PD), and transposed duplicates (TRD) duplications, while singletons accounted for the majority of private genes (Fig. 3H).

### Identification of SVs that may contribute to differences in dihydromyricetin content in vine tea

**Figure 4:**
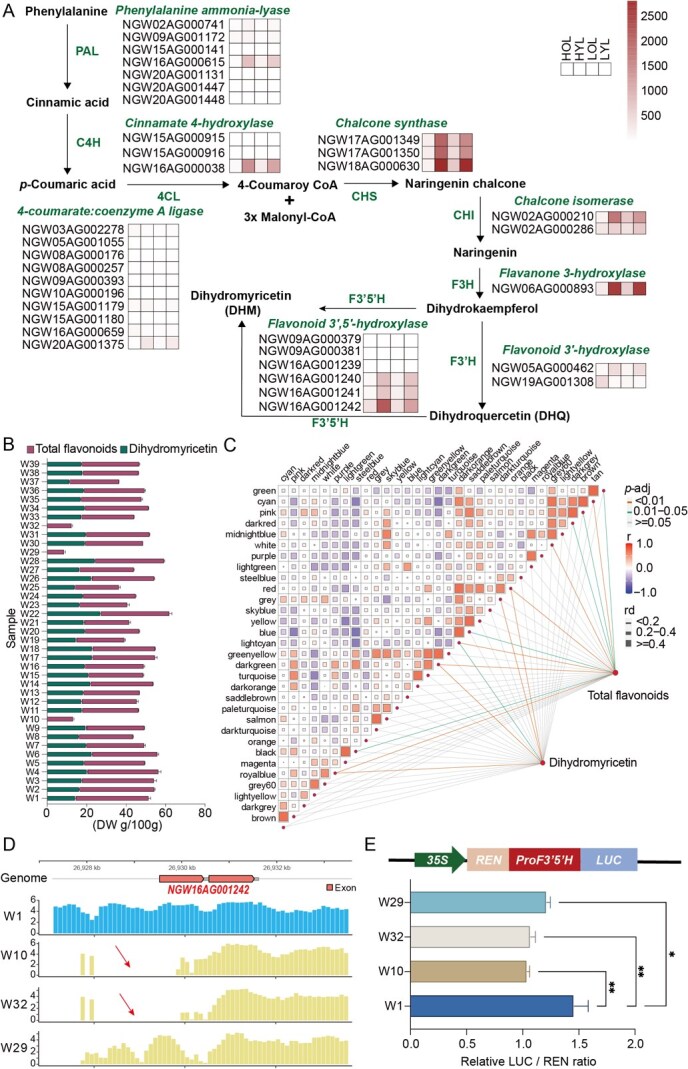
Expression of genes related to the biosynthesis pathway of dihydromyricetin in *N. grossedentata*. (A) Biosynthesis pathway of dihydromyricetin in *N. grossedentata*. The heatmaps illustrate the expression levels in young and old leaves of HY and LY of *N. grossedentata*. (B) Contents of dihydromyricetin and total flavonoids in 39 *N. grossedentata* cultivars. (C) Weighted co-expression network analysis was performed based on the gene expression levels and dihydromyricetin contents of 39 *N. grossedentata* cultivars. Positive and negative correlations are indicated using distinct line patterns, and different gene modules are represented by distinct cluster identifiers rather than colours. (D) The difference map of the promoter and gene body of the enzyme *NgF3*′*5H* between species containing dihydromyricetin and without dihydromyricetin, with red arrows indicating the difference regions. (E) Transient expression experiments showed that different cultivars (W10, W29, and W32) directly reduced the regulatory potential of the promoter on the *F3′5′H* gene

The primary active ingredient of flavonoids in vine teas is dihydromyricetin (DHM), which exerts a variety of effects including the scavenging of free radicals, anti-oxidation, anti-thrombosis, anti-tumor, and anti-inflammatory activities. The biosynthesis of DHM commences with phosphoenolpyruvate and erythrose-4-phosphate, which are produced by photosynthesis. These react further to synthesize naringenin via the shikimic acid pathway, phenylpropanoid metabolic pathway, and flavonoid synthesis pathway. Naringenin is a pivotal intermediate in the synthesis of flavonoids. Subsequent to this, naringenin is converted into dihydrokaempferol by the action of flavanone 3-hydroxylase (F3H), and dihydrokaempferol is then further hydroxylated to dihydromyricetin by the action of flavanol synthase (FLS). Previous studies have reported relatively high levels of active ingredients in young leaf samples in vine tea [15], so we analyzed the expression of DHM biosynthesis-related genes in young and old leaves for exploring the key genes in the biosynthesis pathway of DHM (Fig. 4A). The expression trends of *PAL* (*NGW16AG000615*), *C4H* (*NGW16AG000038*), three *CHS* (*NGW17AG001349*, *NGW17AG001350*, *NGW18AG000630*), *CHI* (*NGWO2AG000210*), *F3H* (*NGWO6AG000893*), and *F3′5′H* (*NGW16AG001242*) genes exhibited a congruence with the content of DHM in leaves, with the content in young leaves demonstrating higher levels than those in old leaves (Fig. 4A).

To explore the reasons for the differences in DHM in different cultivars, the DHM and total flavonoid (FLA) contents of 39 vine tea cultivars were examined. The results showed that cultivars W10 (DHM: 0.25 g/100 g; FLA: 13.17 g/100 g), W29 (DHM: 0.05 g/100 g; FLA: 8.8 g/100 g), and W32 (DHM: 0.07 g/100 g; FLA: 12.57 g/100 g) had the lowest DHM content, whereas the highest content was found in W22 (DHM: 27.1 g/100 g; FLA: 35.2 g/100 g) (Fig. 4B, Table S5). The leaves transcriptomes of 39 cultivars were sequenced, gene co-expression networks were constructed, and correlations between co-expressed gene modules and detected metabolite content were assessed. The five modules were found to be significantly correlated with DHM content: modules of red, grey, darkgreen, royalblue, and light cyan were correlated (*P* < 0.05) (Fig. 4C). We further explored the number of transcription factors in these modules, with the highest percentage of bHLH, MYB, and WRKY families, which have been reported to have key roles in the plant flavonoid synthesis pathway (Fig. S9 and Table S7). This analysis provides a list of TF candidates to be considered in DHM biosynthesis.

To further explore the genetic basis underlying the differences in DHM content among cultivars, we aligned the resequencing data to the previously constructed pan-genome reference and extracted representative SVs larger than 50 bp, including duplication (DUP), translocations (TRANS), deletions (DEL), and inversions (INV). The number of SVs identified in individual cultivars ranged from 27 732 to 125 439 (Table S6). We then systematically examined SVs in genes encoding key enzymes involved in the dihydromyricetin (DHM) biosynthetic pathway. Notably, we detected a 1038-bp deletion in the promoter–first exon region of *NgF3′5′H* (*NGW16AG001242*) in both W10 and W32 cultivars (Fig. 4D), which may affect the transcriptional regulation of this gene. Furthermore, we examined the expression of genes related to DHM biosynthesis in all samples and found that *NgF3′5′H* (*NGW16AG001242*) expression was significantly reduced in both W10 and W32 cultivars (Fig. S10A). We conjectured that this might be one of the reasons for the low DHM content in these two cultivars. The deletion of the promoter and the first exon may have resulted in low expression levels of this gene. F3′5′H has been reported to be a key enzyme in the conversion of dihydroquercetin (DHQ) to DHM in previous studies [18]. We cloned the gene into an expression vector and observed that it belonged to the cell membrane localization by injecting tobacco leaves, while leaves expressing NgF3′5′H were subjected to enzyme activity examination, and it was found that NgF3′5′H was able to promote the synthesis of DHM from DHQ (Fig. S10B), which indicates that the gene has a catalytic function. Using the website of protein structural domains (http://smart.embl-heidelberg.de/) to predict the structural domain of NgF3′5′H, we found that SV resulted in the absence of a transmembrane structural domain of NgF3*′*5*′*H in W10 and W32. Further, we cloned *NgF3′5′H* in W1 and *ΔNgF3′5′H* in W32 into expression vectors and observed their transiently transformed Arabidopsis protoplasts using confocal microscopy, and found that *ΔNgF3′5′H* could not be correctly localized to the membrane (Fig. S10C). Through the dual-luciferase (LUC) assay, we tested the promoter activity of various F3*′*5*′*H cultivated in W1, W10, W29, and W32, and found that the promoter activity of W10, W29, and W32 was significantly lower than that in W1(Fig. 4E), indicating that the loss of the promoter region may be a factor causing the differential expression of DHM in different cultivars.

## Discussion

*Nekemias grossedentata* (commonly known as vine tea) is an important traditional herbal beverage widely consumed in China, renowned for its rich flavonoids content and diverse pharmacological properties, such as anti-atherosclerotic, lipid-lowering, and hypoglycemic effects [19–22]. While previous studies based on transcriptome assemblies identified a large number of unigenes in *N. grossedentata* [23], the lack of a reference genome has limited deeper insights into its evolutionary history and metabolic specialization. Here, we performed high-quality chromosome-level genome assemblies for two *N. grossedentata* accessions using PacBio HiFi long reads in combination with Hi-C data, providing the first comprehensive genomic resource for this medicinal species (Table 1). The high contiguity and accuracy of the assemblies, together with the construction of a pan-genome based on 39 resequenced *N. grossedentata* accessions, offer a valuable foundation for exploring the phenotypic diversity of this species.

From a taxonomic and evolutionary perspective, the traditional genus *Ampelopsis* has been subdivided into two genera: *Ampelopsis* s.s. and *Nekemias*. The three most commonly used vine tea varieties—*N. grossedentata*, *N. cantoniensis*, and *N. megalophylla*—are all classified under *Nekemias* [3]. Our results further clarify the position of *Nekemias* within Vitaceae, highlighting its divergence from *C. rotundifolia* and *V. vinifera* around 15.36–38.57 MYA and 8.53–27.24 MYA. Notably, no recent whole-genome duplication (WGD) events were detected in *N. grossedentata* (Fig. 2A). Moreover, its genome size (596–607 Mb) is substantially larger than that of *C. rotundifolia* (350.69 Mb) and *V. vinifera* (475 Mb), likely due to a recent burst of LTR retrotransposon activity around 0.5 MYA (Fig. S5D). Importantly, comparative analyses among the four haplotype-resolved assemblies revealed that the major genomic differences across haplotypes are also largely attributable to variation in LTR content and distribution (Fig. S5E). This indicates that transposon-mediated genome remodeling has not only shaped the overall genome size of *N. grossedentata* but also contributed to haplotype-level diversity, which may in turn influence allelic variation in metabolic genes.

The dynamic nature of the *N. grossedentata* genome has important consequences for genetic diversification and metabolic adaptation. Population genetic analyses of 39 cultivated accessions revealed four distinct groups with clear geographic patterns (Fig. 3 and Fig. S8). Groups 1 and 3 clustered closely with low FST values and overlapping PCA signals, whereas Groups 2 and 4 exhibited higher differentiation and Δπ contrasts. Although these indices are traditionally applied to natural populations, their patterns here likely reflect historical breeding, local cultivation practices, and human-mediated selection rather than purely natural evolutionary processes. Migration analyses further suggested that Groups 2 and 4 served as major hubs of germplasm exchange, while Groups 1 and 3 acted as secondary links between central and southern production regions (Fig. S8A and C). This framework provides insights into how human-driven propagation and selection have shaped the genomic diversity of vine tea cultivars.

Such genome-wide and population-level variation has been widely reported to be closely associated with the regulation of secondary metabolic pathways [24, 25]. The haplotype-level differences mediated by transposable elements and population-specific structural variants may affect the regulation and allelic diversity of key metabolic genes, thereby contributing to variation in bioactive compounds. Flavonoid 3*′*,5*′*-hydroxylase (F3′5′H) is a heme-containing oxidase that plays a key rate-limiting role in flavonoid biosynthesis, especially in the production of DHM. Our study is the identification of NgF3′5′H, a key enzyme involved in DHM biosynthesis (Fig. 4A and Fig. S9B). Through integrative analyses of structural variants and metabolite content, we found that structural variation in the *NgF3′5′H* gene is likely to affect DHM accumulation in different accessions (Fig. 4D). Structural variation affecting NgF3′5′H appears to underlie differences in DHM accumulation among accessions, providing a potential explanation for metabolite diversity across varieties. Moreover, tandem duplication of this gene likely enhances its catalytic efficiency compared with homologs in other Vitaceae species. Such genomic features may partly account for the unusually high levels of DHM in vine tea and illustrate how structural variation can fine-tune specialized metabolism.

## Materials and methods

### Plant materials

The source material used to sequence the *N. grossedentata* genome was from the Baiyun Base of Guangdong Academy of Agricultural Sciences, China (23°23*′*14.1*′′*N 113°26*′*15.6*′′*E). A total of 39 *N. grossedentata* accessions were used in this study. These accessions were originally collected by the Guangdong Academy of Agricultural Sciences directly from local producers and farmers across four provinces in southern China, including Hunan, Guangdong, Guangxi, and Jiangxi. To ensure uniform growth conditions and long-term conservation, all collected accessions were subsequently transplanted into the germplasm nursery of the Guangdong Academy of Agricultural Sciences (Guangzhou, China), where they have been maintained under standard horticultural management. Detailed information on the sampling locations and sources of each accession is provided in Table S3.

After the materials were obtained, they were placed in liquid nitrogen and frozen at −80°. Plant DNA was extracted using the Tiangen Polysaccharide and Polyphenol Plant Genomic DNA Extraction Kit (DP360). Plant RNA was extracted using the Tiangen RNAprep Pure Polysaccharide and Polyphenol Plant Total RNA Extraction Kit (DP441).

### Flow cytometry and *k*-mer analysis

The genome size of the target plants was determined by means of flow cytometry. The internal reference was tomato and maize. B73 seeds were obtained from the Kunming Institute of Botany, Chinese Academy of Sciences, and the experimental materials were young leaves one month after seed germination. The tissues were rapidly minced using a sharp blade and then placed on ice for 10 minutes in the dissociation solution. Following this, the samples were filtered through a 40-μm pore size filter to obtain the cell nucleus suspension. Pre-cooled propidium iodide and an appropriate volume of RNAase solution in an appropriate volume of cytosolic suspension were placed on ice and protected from light for staining for 0.5 to 1 hour [26]. Tomato and maize B73 were used as the internal reference, and the suspensions of the samples to be tested and the suspensions of the internal reference samples were mixed in the appropriate proportions. The stained cell nucleus suspension samples were then examined using a BD FACScalibur flow cytometer with 488 nm blue light excitation to detect the fluorescence intensity of the emitted light of propidium iodide, and 10 000 particles were collected for each assay. The coefficient of variation CV% was controlled within 5%. Graphical analysis was performed using Modifit 3.0 analysis software.

The *k*-mer analysis method was implemented as follows: *k*-mer calculations were performed on the second-generation sequencing data using Jellyfish (v2.3.0) [27], and the generated results were visualized using GenomeScope 2.0 [28].

### Genome sequencing and assembly

For the third-generation long-read sequencing, the PacBio Revio platform of Berry Genomics was used for sequencing, and data with a quality value greater than or equal to Q20 were extracted from the CCS Data. For the Hi-C sequencing experiment, the library was prepared with the Dpn II restriction endonuclease and sequenced on the Illumina NovaSeq platform of Berry Genomics, and data with a base quality less than <15 were removed. Transcriptome sequencing was performed on the HiSeq 2500 platform of Berry Genomics.

The processing of HiFi data was undertaken using PBccs (v6.4.0) software (https://github.com/PacificBiosciences/ccs), with subsequent preliminary assembly conducted via Hifiasm (v0.19.7) [29] software (in Hi-C fixed-phase mode). Following the acquisition of the genome sketch, the resulting stereotyped haplotype-resolved genomes were merged using the 3D-DNA pipeline[30], with further anchoring to the chromosomes (−r 0; non–error-correcting mode), and finally adjusted using the Juicebox (v1.11.08) software [30] to obtain the final genome.

Genomic integrity was assessed based on the consistency of the sequencing data *k*-mer and the assembly result *k*-mer, and QV calculation was performed by merqury software [31]. LAI metrics were assessed based on the LTR of the genome for continuity. The LTR prediction of the genome was performed using ltrharvest software [32], and then the LTR prediction was performed using LTR_FINDER software [33], and after combining the two results, LTR_retriever software [34] was used to integrate them and calculate LAI.

### Genomic gene and TE prediction, functional annotation

The subsequent annotation of genes was facilitated by the utilization of both transcriptome data and repeat-masked genomes. This approach involved the integration of ab initio prediction, homologue-based prediction, and transcriptome-based prediction, resulting in the prediction of gene structures. The comparison of transcriptome data was conducted using HISAT2 (v2.21) software[35], and all transcripts obtained were validated through the use of PASA pipeline (v2.5.1) software[36]. Following this validation process, the transcripts were integrated using Augustus (v3.4.0) software[37]. Finally, MAKER (v3.01.03) was used to obtain annotations of gene structures[37]. The correction of gene structure annotation is facilitated by the SynGAP tool[38]. Gene functional annotation (selection of plant specific databases) was performed using eggnog-mapper [39](http://eggnog-mapper.embl.de/).Transposon factors (TE) were annotated using EDTA (v2.1.0) software[40] and DeepTE (v2.2.4) software[41].

### SV detection and validation

SV detection among haplotype-resolved assemblies: Pairwise genome alignments of the four haplotype-resolved assemblies were performed using minimap2 v2.24 with default parameters. Structural variants were then identified with SyRI v1.5 [16] to detect insertions, deletions, inversions, duplications, and translocations across the haplotypes. The methodology of the published articles was referred to for the purpose of detection[17], and the structural variants were re-detected through the SyRI process with default parameters once more. The structures were then counted, and finally, a comparison was performed using the Integrative Genomics Viewer [42].

SV detection based on resequencing data: Clean short-read resequencing data of 39 accessions were mapped to the pangenome reference genome constructed through iterative assembly using BWA-MEM v0.7.17 with default settings. Structural variants were called using LUMPY v0.2.13 [43], and high-confidence SVs were retained after filtering. Representative SVs were further validated and visualized using IGV [42].

### Phylogenetic tree construction and divergence time calculation

Phylogenetic trees were constructed using the published genomes of 11 species. The construction of species trees was facilitated by the utilization of OrthoFinder (v2.5.3) software [44], which was employed to execute direct homology analyses on the protein sequences of all species. Subsequent to this, inter-species divergence time calculations were performed using the mcmctree tool in the PAML (v4.10.7) software[45] package (tree root ages were calibrated from the TimeTree database). Shrinking expanded gene families were analyzed by CAFE (v5.1.0) software[46]. GO and KEGG analyses were performed using the clusterProfiler of R package[47].

### Resequencing analysis and population genetic analyses

The quality of the raw sequencing data was assessed using FastQC (v0.11.8), after which low-quality base and splice sequences were removed using Trimmomatic (v0.36). The high-quality reads that passed the quality control were then compared to the reference genome (NGW-HapA) using BWA-MEM (v0.7.18) to generate SAM-format files. The results of this comparison were then compared by samtools (v1.6) and Picard (v2.25.7) processing, including conversion to BAM format, sorting, labeling, and removal of duplicate reads. This was done to reduce the impact of PCR amplification bias on subsequent analysis. Subsequently, the HaplotypeCaller of GATK4 (v4.2.2.0) was utilized for variant detection, preceded by the completion of the steps of base quality score recalibration (BQSR) and insertion deletion (Indel) recalibration. The variants were filtered using a rigid A hard filtering strategy, with commonly employed thresholds including SNP QD > 2.0, FS < 60.0, MQ > 40.0, etc. The detected variants were functionally annotated using ANNOVAR.

Population structure was inferred from high-quality SNPs using ADMIXTURE (v1.3.0) with cross-validation to determine the optimal K value. Principal component analysis (PCA) was performed using PLINK (v1.9), and a neighbor-joining tree was constructed with PHYLIP (v3.697). Nucleotide diversity (π) was calculated for each group using VCFtools (v0.1.17) with a 50-kb sliding window and 10-kb step size. Gene flow and historical relationships among groups were analyzed using TreeMix (v1.13), allowing for up to three migration edges to be tested.

### Pan-genome construction and analysis

In the pan-genome analysis based on iterative assembly, starting from the reference genome, the sequencing data of each sample were sequentially aligned to the reference genome. Unaligned sequences were then extracted and assembled, and the reference genome was merged and updated. The final pan-genome was then obtained after iteration for each sample. The final pan-genome was assembled by using Soapdenovo2 (r240) software[48], the unmatched and partially matched contigs were extracted by Quast (v5.3.0) software[49], and the redundancy was removed by clustering with cd-hit (v4.8.1) software[50]. SGSGeneLoss (v0.1) software[51] was then utilized to calculate the gene coverage based on the second-generation sequencing data of each individual, and the gene family PAV information was obtained by judging the presence or absence of genes according to the set coverage threshold. Finally, the gene family PAV information was obtained by clustering the gene families with Orthofinder software and integrating the core and variable gene families.

### Detection of dihydromyricetin and total flavonoids

The content of dihydromyricetin and total flavonoids in vine tea leaf slices was detected using a liquid chromatograph LC1260. The following protocol was employed for sample preparation: vine tea leaves were dried and crushed to a uniform powder state. Then, 0.2 g of the sample was accurately weighed, added to 20 ml of 70% ethanol solution, and extracted by ultrasonic extraction for 30 minutes. The mixture was then subjected to centrifugation at 4000 rpm for 10 minutes, after which the upper layer was collected and stored for future use. The detection process is outlined as follows: An Agilent LC1260 high performance liquid chromatograph equipped with a diode array detector (DAD) was utilized, and the column was Agilent ZORBAX Eclipse Plus C18 (4.6 × 250 mm, 5 μm). The mobile phase was a solution of methanol and 0.2% phosphoric acid, eluted through the column at a flow rate of 1.0 ml/min. The detection wavelengths were 290 nm for dihydromyricetin and 360 nm for total flavonoids, with an injection volume of 10 μl at a temperature of 30°C. The content of dihydromyricetin was calculated from the standard curve prepared by using dihydromyricetin standard, and the content of total flavonoids was obtained by the aluminium-salt colorimetric conversion method using rutin as the standard. The total flavonoid content was then calculated by utilizing the standard curve of the dihydromyricetin standard. The experiment was repeated on three occasions, and the results were averaged.

### Identification of genes in the dihydromyricetin biosynthetic pathway

Based on the protein sequences and Pfam structural domains of genes reported previously for each catalytic step [23], genes involved in the synthesis of dihydromyricetin in vine tea were identified using BLAST (v2.14.1) and HMMER (v3.4). For BLAST searches, we used an e-value threshold of 1e−5, with identity ≥40% and coverage ≥50%. For HMMER, sequences were considered significant with a domain E-value ≤1e−3. The results from BLAST and HMMER were then integrated, and conserved structural domains were further verified by comparison with genes in other species to construct a phylogenetic tree.

### Gene expression and enzyme activity assays

The target gene was then cloned into the binary expression vector *Pcambia1300221* with a green fluorescent protein (GFP) tag,using primers listed in Table S8. A comparison was made with the reference sequence by means of sequencing. The constructed vector was then transformed into tobacco leaves by means of the injection method using Agrobacterium rhizogenes. The expression was then observed by confocal microscopy. The injected leaves were then ground in liquid nitrogen, formaldehyde was added, and the samples were placed in a sonicator at 45° for 15 minutes. The obtained solution was subjected to centrifugation, after which the resulting pellet was assayed in liquid chromatography.

### Protoplast extraction and detection

The term ‘Arabidopsis protoplast method’ refers to a series of published papers[52] in which genes with different truncations were cloned into transient expression vectors. Following transformation into protoplasts, the subcellular localization of the genes was observed by confocal microscopy.

## Supplementary Material

Web_Material_uhaf307

## Data Availability

The raw genome sequence and raw transcriptome sequencing data are available in China National center for Bioinformation under Bioproject ID: PRJCA045905. Genome assembly and annotation are available in Figshare link: 10.6084/m9.figshare.30136549.v3. The RNA-seq data from PRJNA549404 were also incorporated to support transcriptome analyses.
