# Supplementary figures and images for: Pan-genome assembly of vine tea (Nekemias grossedentata) reveals structural variation in its dihydromyricetin biosynthesis diversity
Source: Hortic Res. 2025 Nov 18;13(2):uhaf307. doi: 10.1093/hr/uhaf307 (PMC12936442; doi:10.1093/hr/uhaf307)

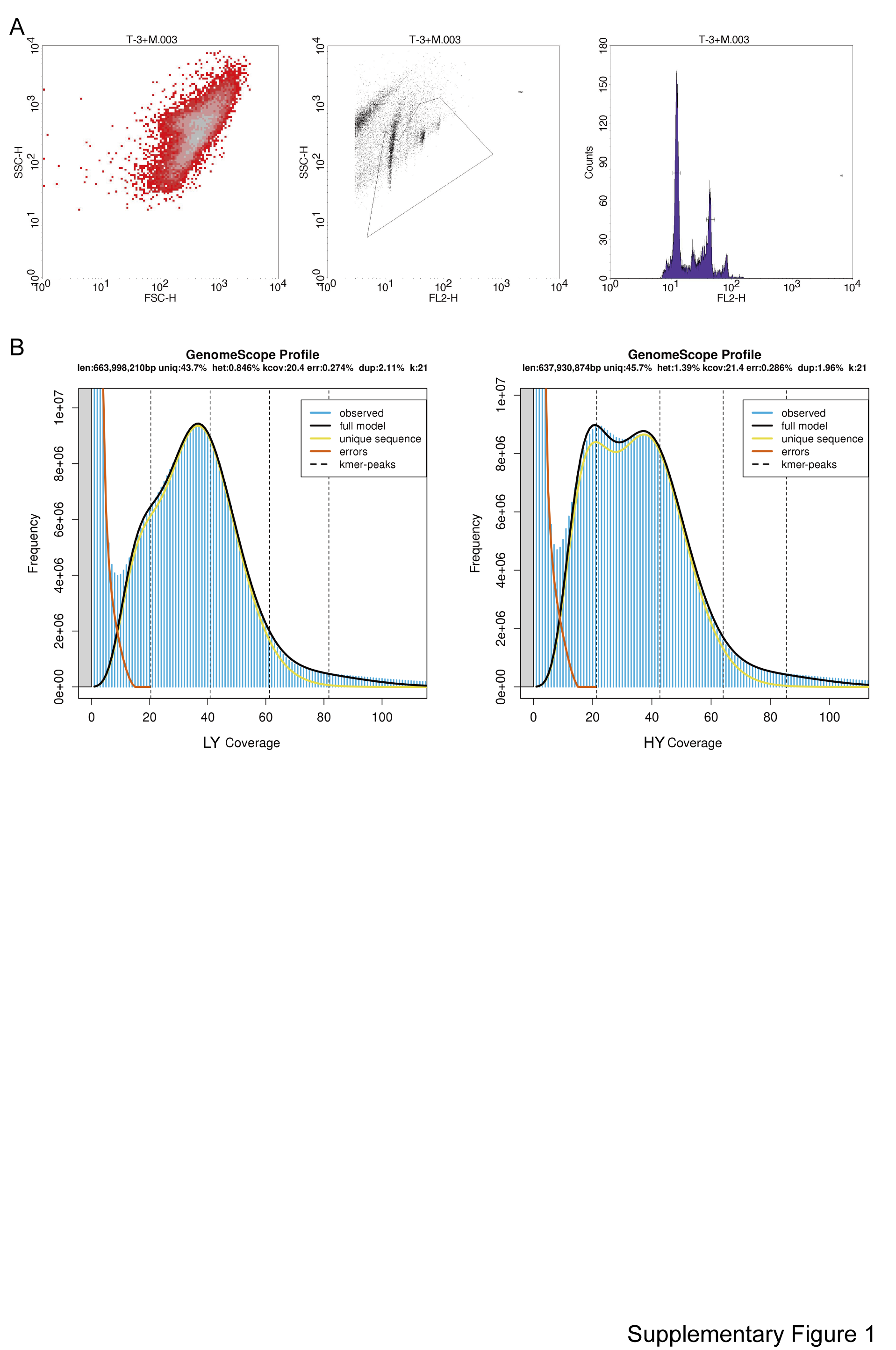

Supplement: Web_Material_uhaf307 [file web_material_uhaf307.zip › Supplementary Figure 1.png]

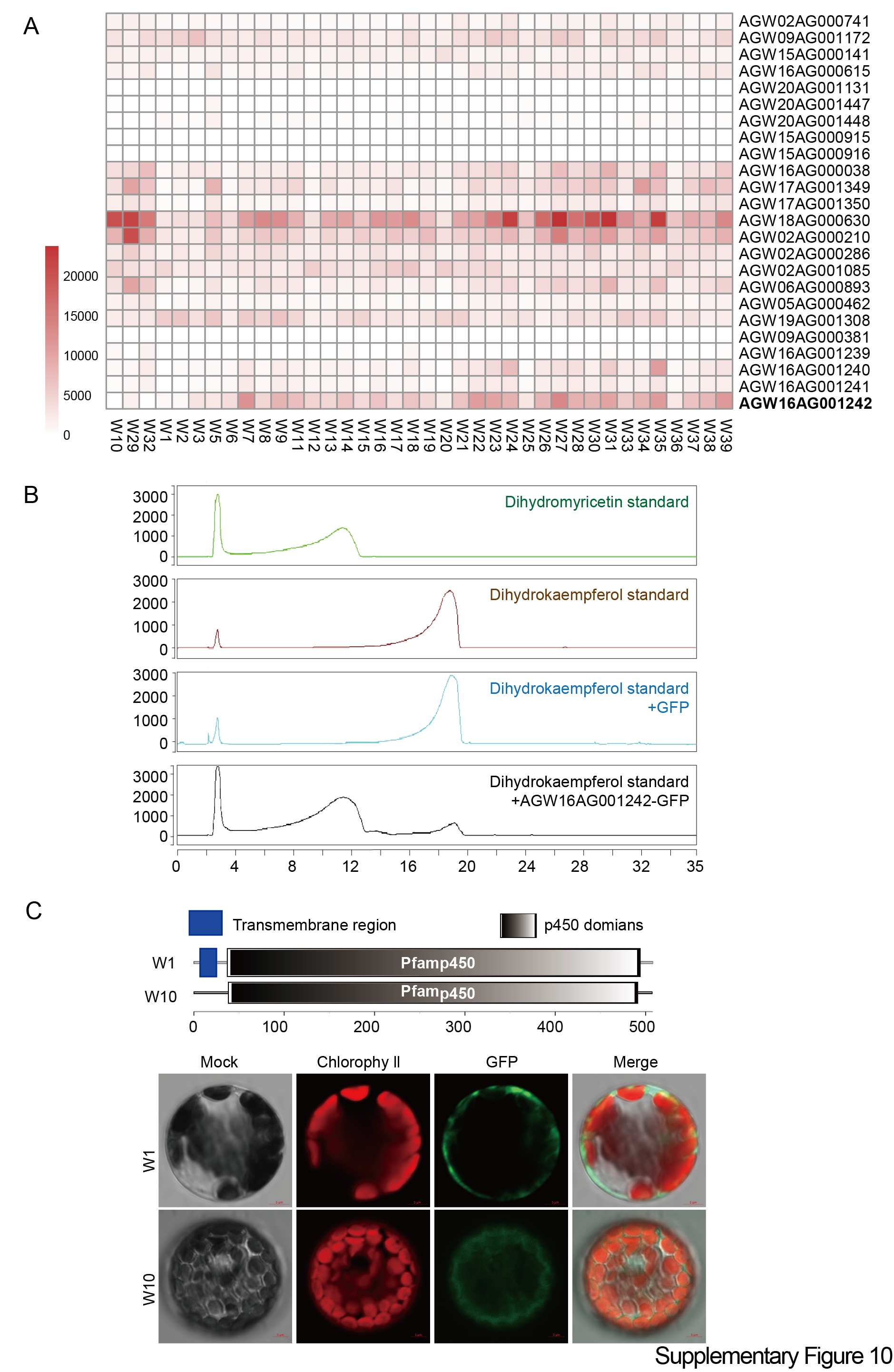

Supplement: Web_Material_uhaf307 [file web_material_uhaf307.zip › Supplementary Figure 10.png]

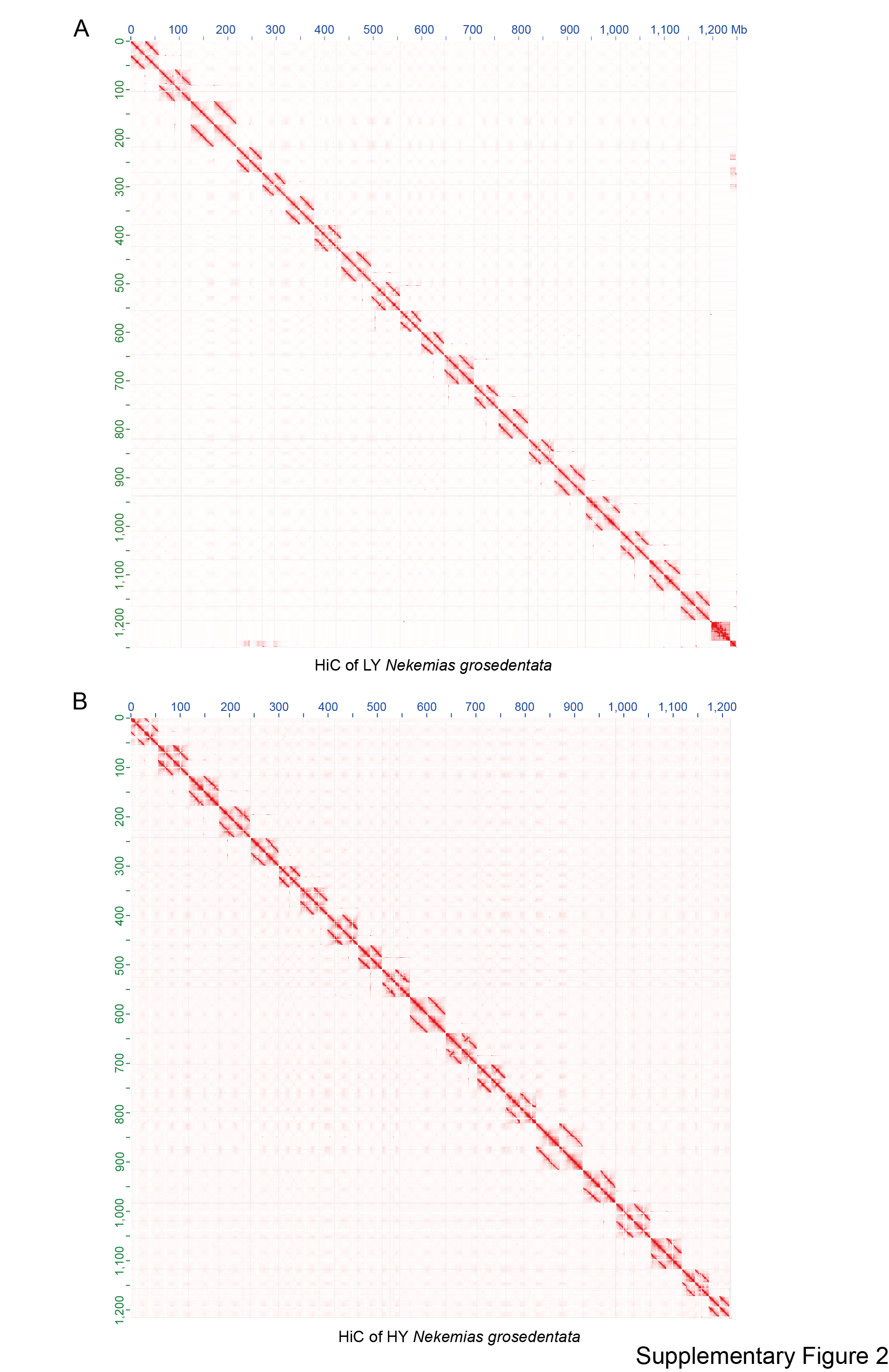

Supplement: Web_Material_uhaf307 [file web_material_uhaf307.zip › Supplementary Figure 2.png]

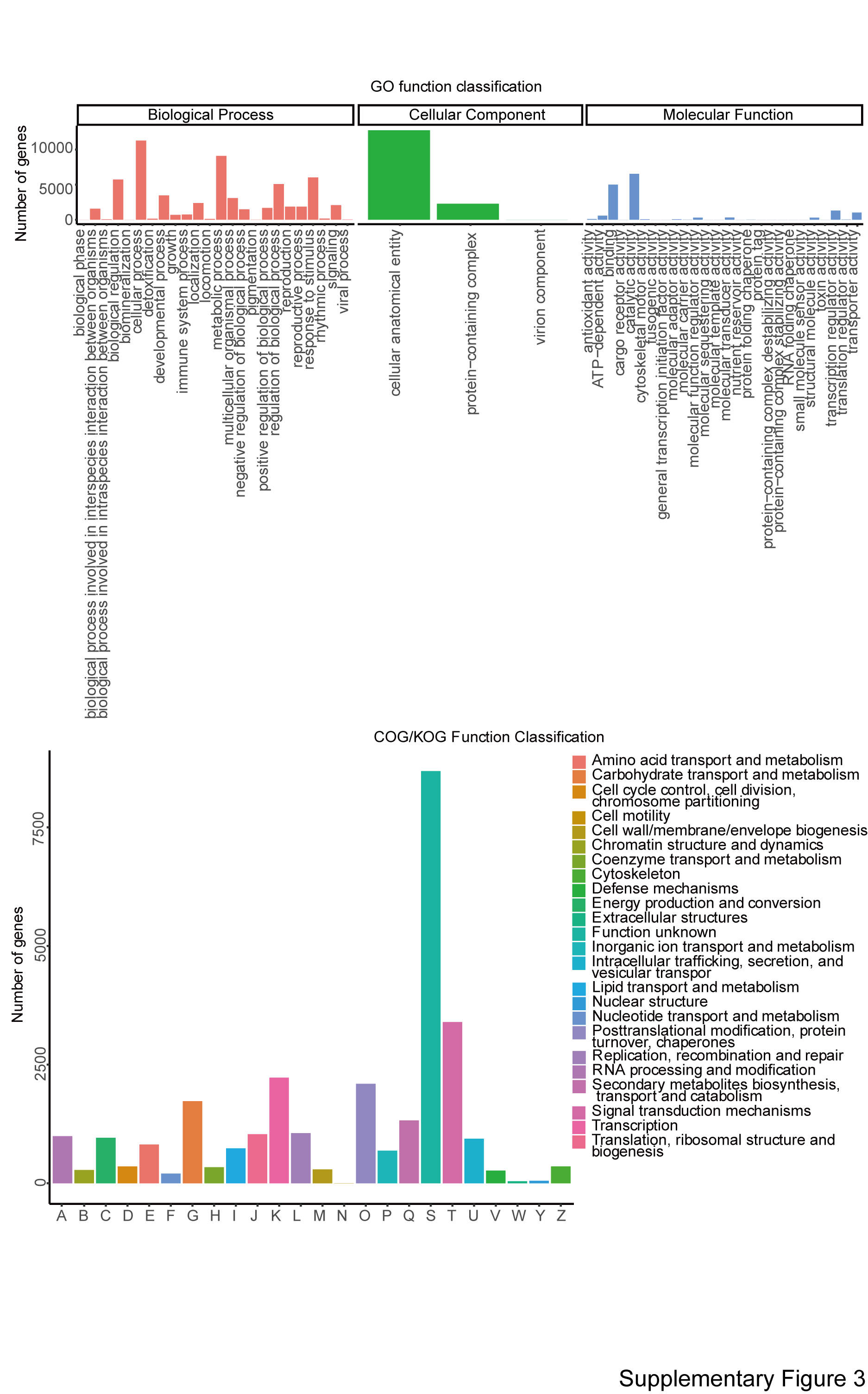

Supplement: Web_Material_uhaf307 [file web_material_uhaf307.zip › Supplementary Figure 3.png]

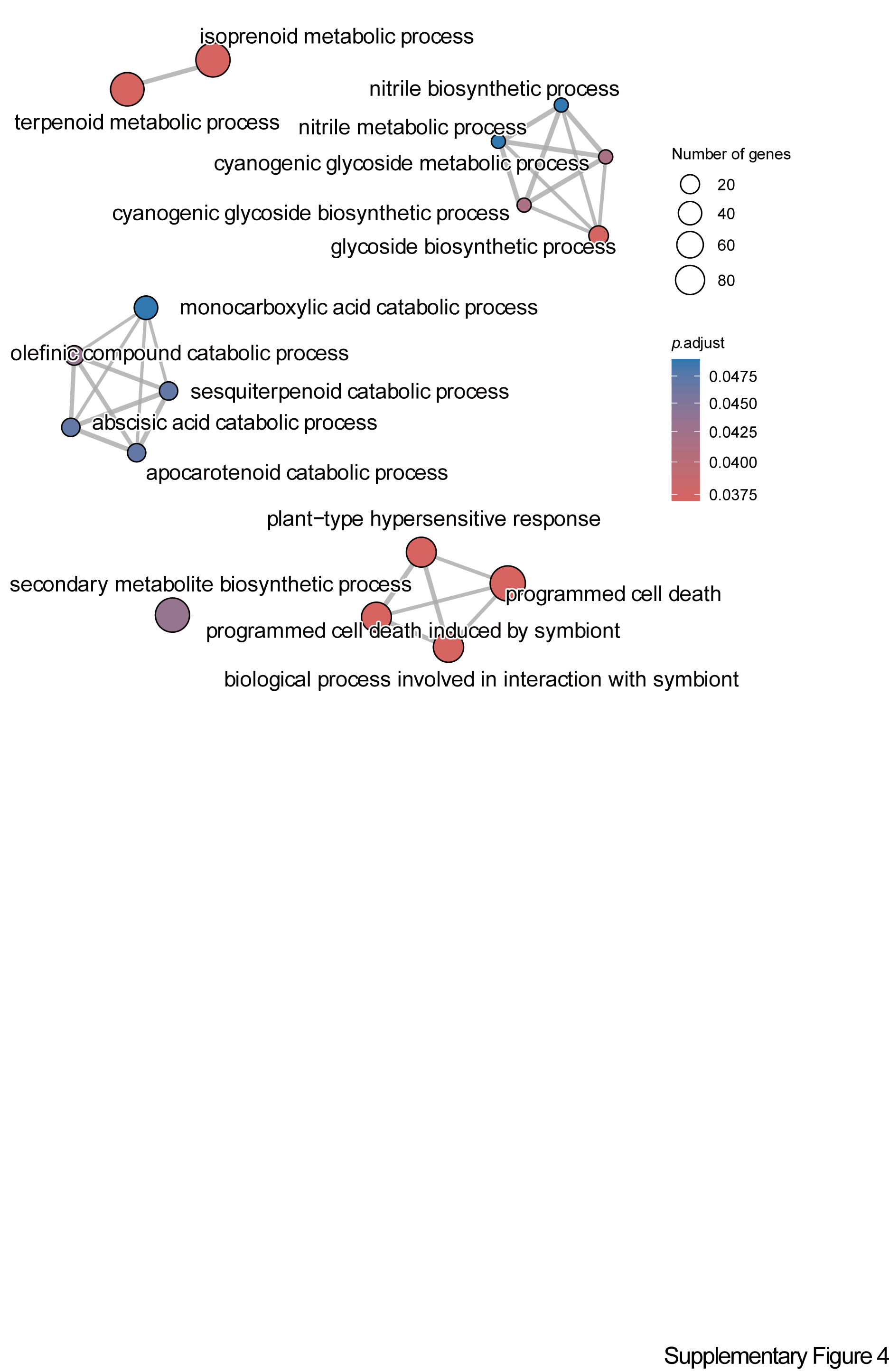

Supplement: Web_Material_uhaf307 [file web_material_uhaf307.zip › Supplementary Figure 4.png]

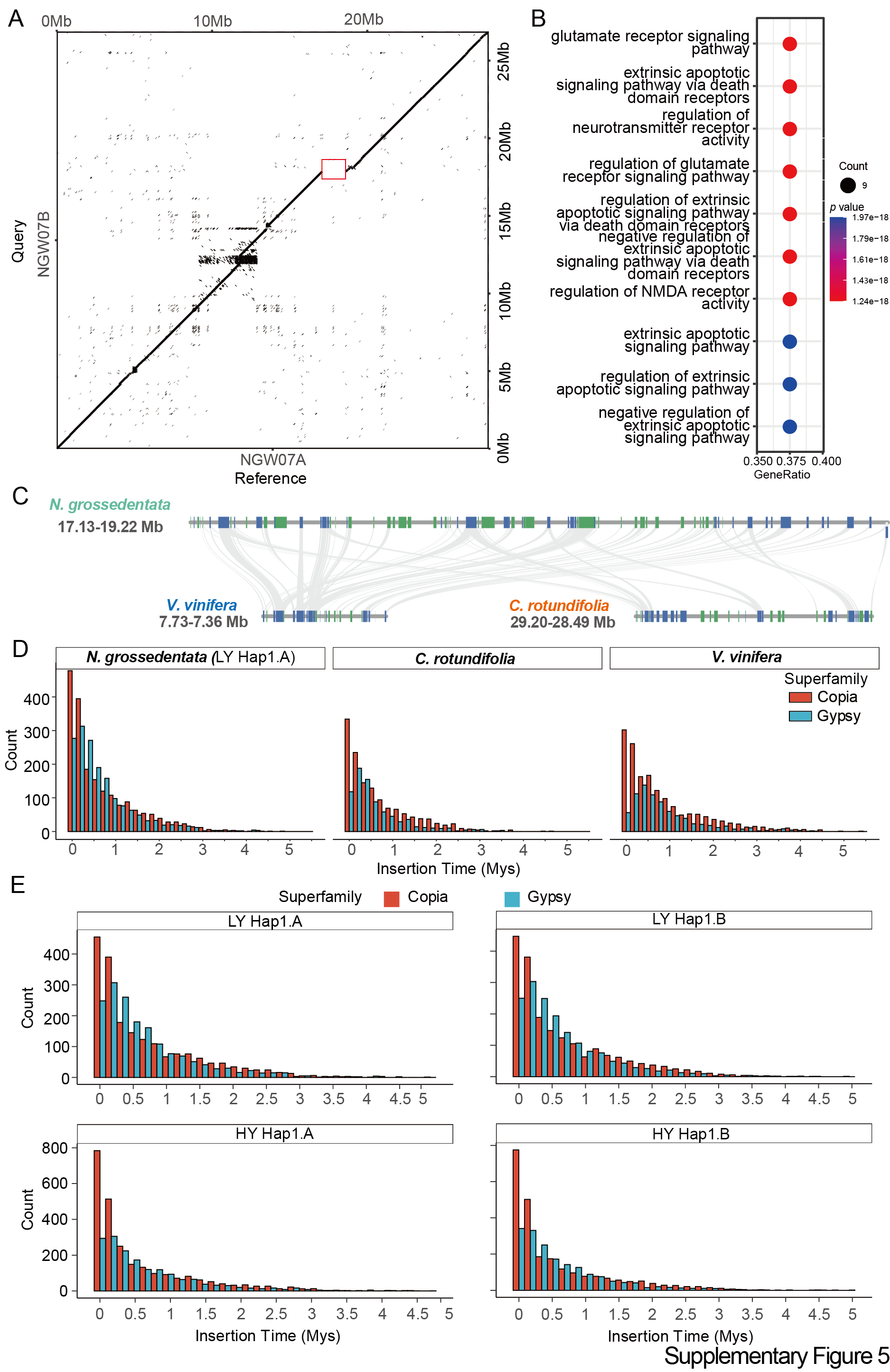

Supplement: Web_Material_uhaf307 [file web_material_uhaf307.zip › Supplementary Figure 5-1.png]

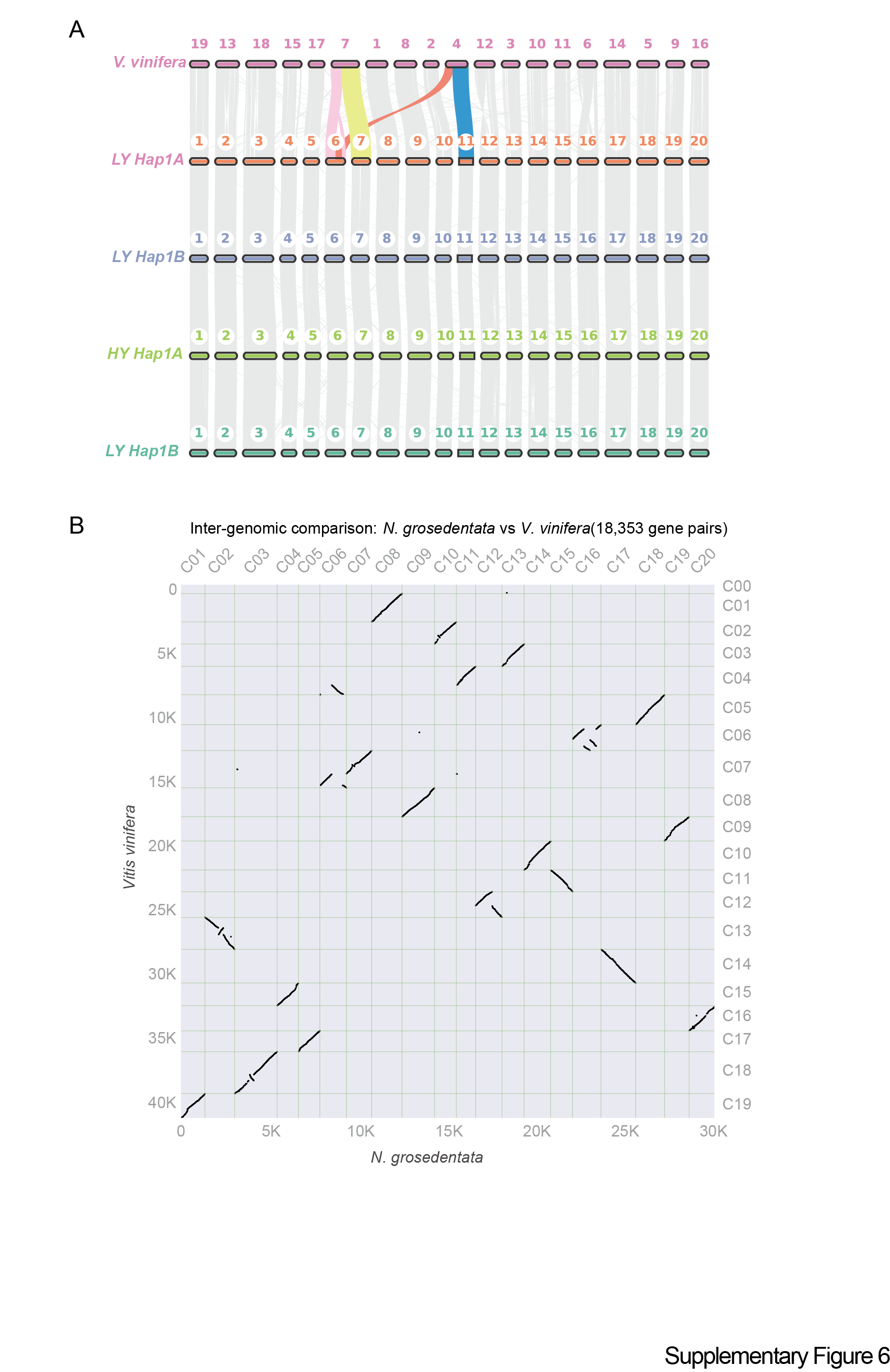

Supplement: Web_Material_uhaf307 [file web_material_uhaf307.zip › Supplementary Figure 6.png]

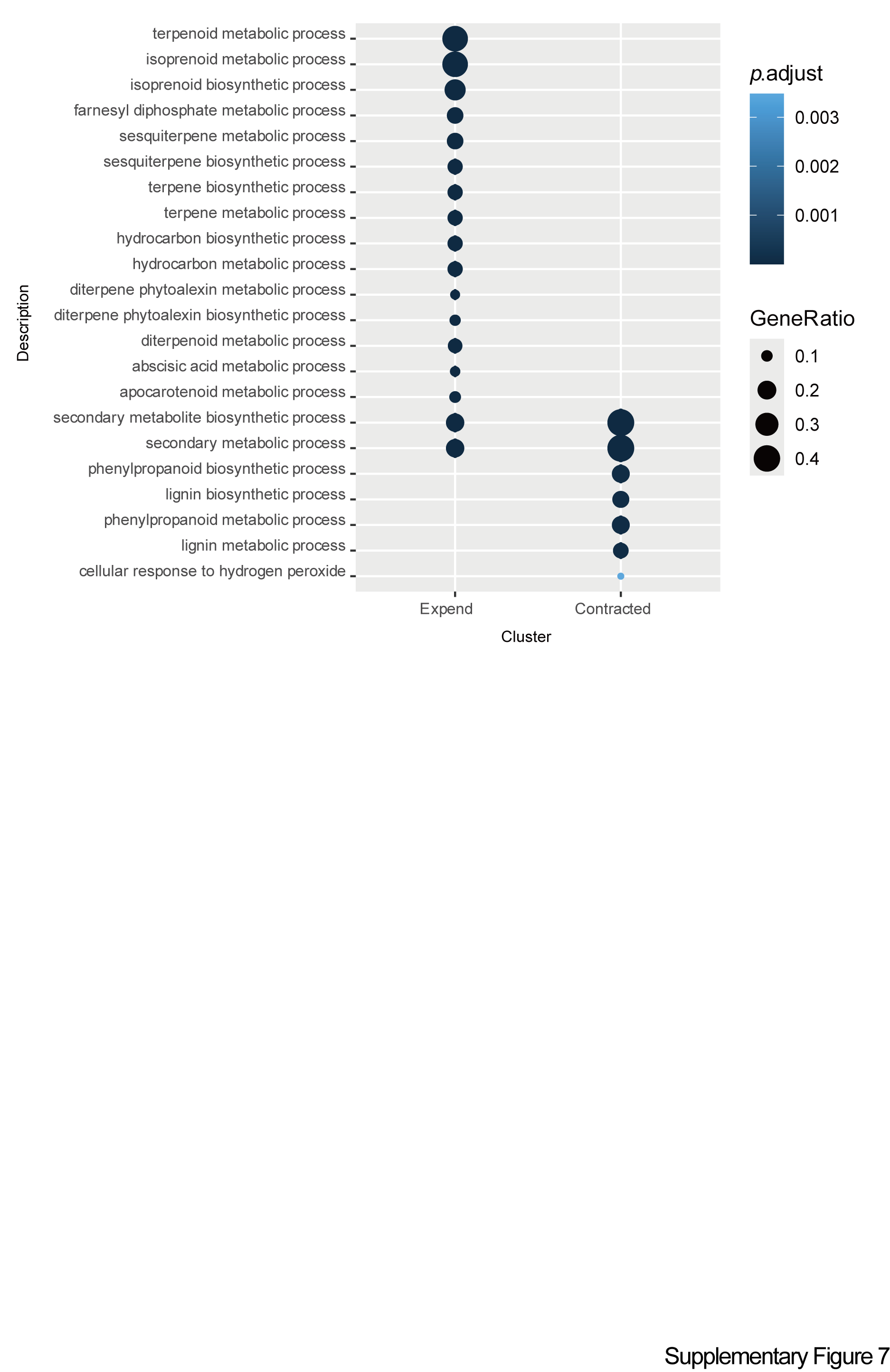

Supplement: Web_Material_uhaf307 [file web_material_uhaf307.zip › Supplementary Figure 7.png]

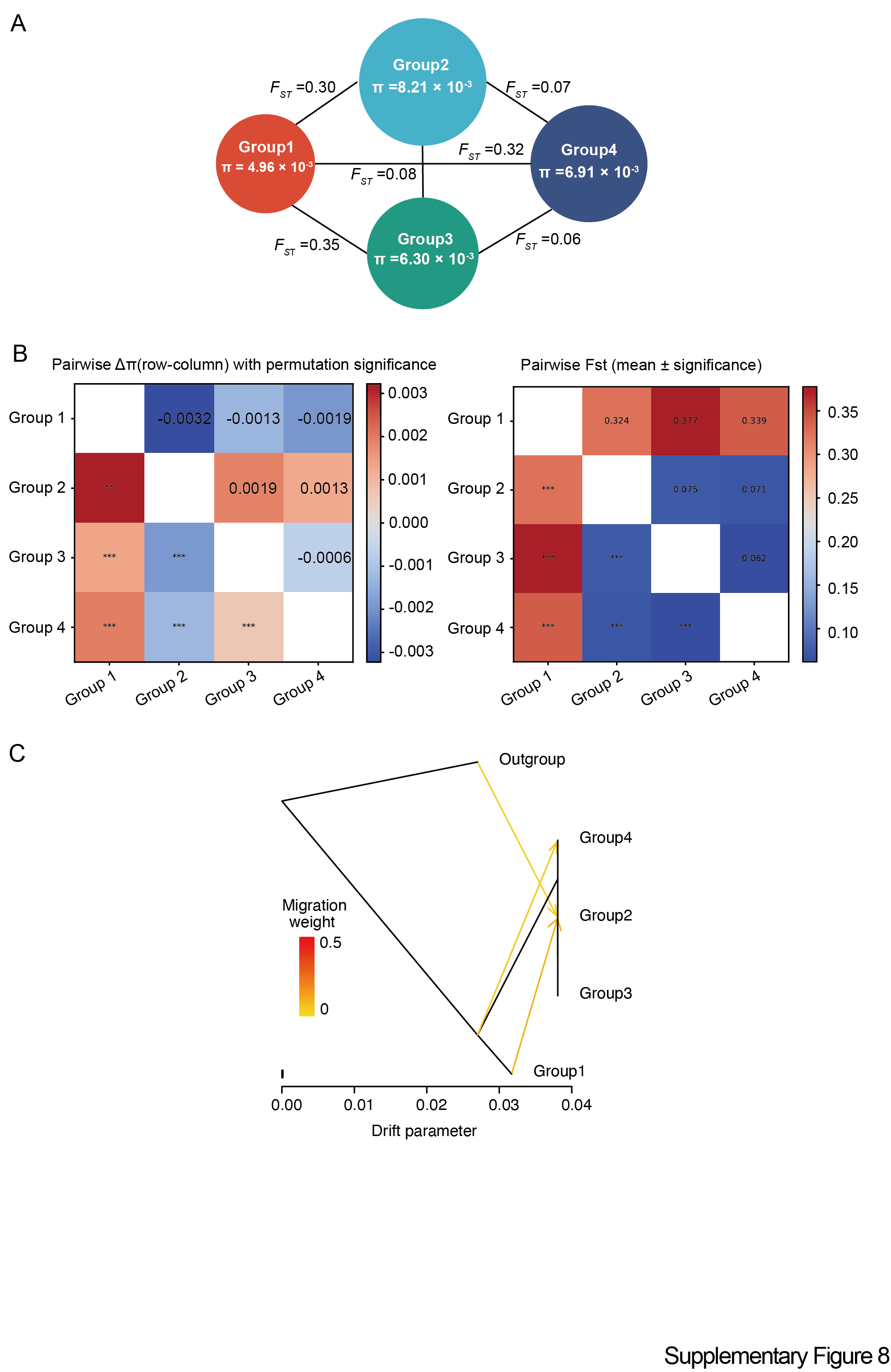

Supplement: Web_Material_uhaf307 [file web_material_uhaf307.zip › Supplementary Figure 8.png]

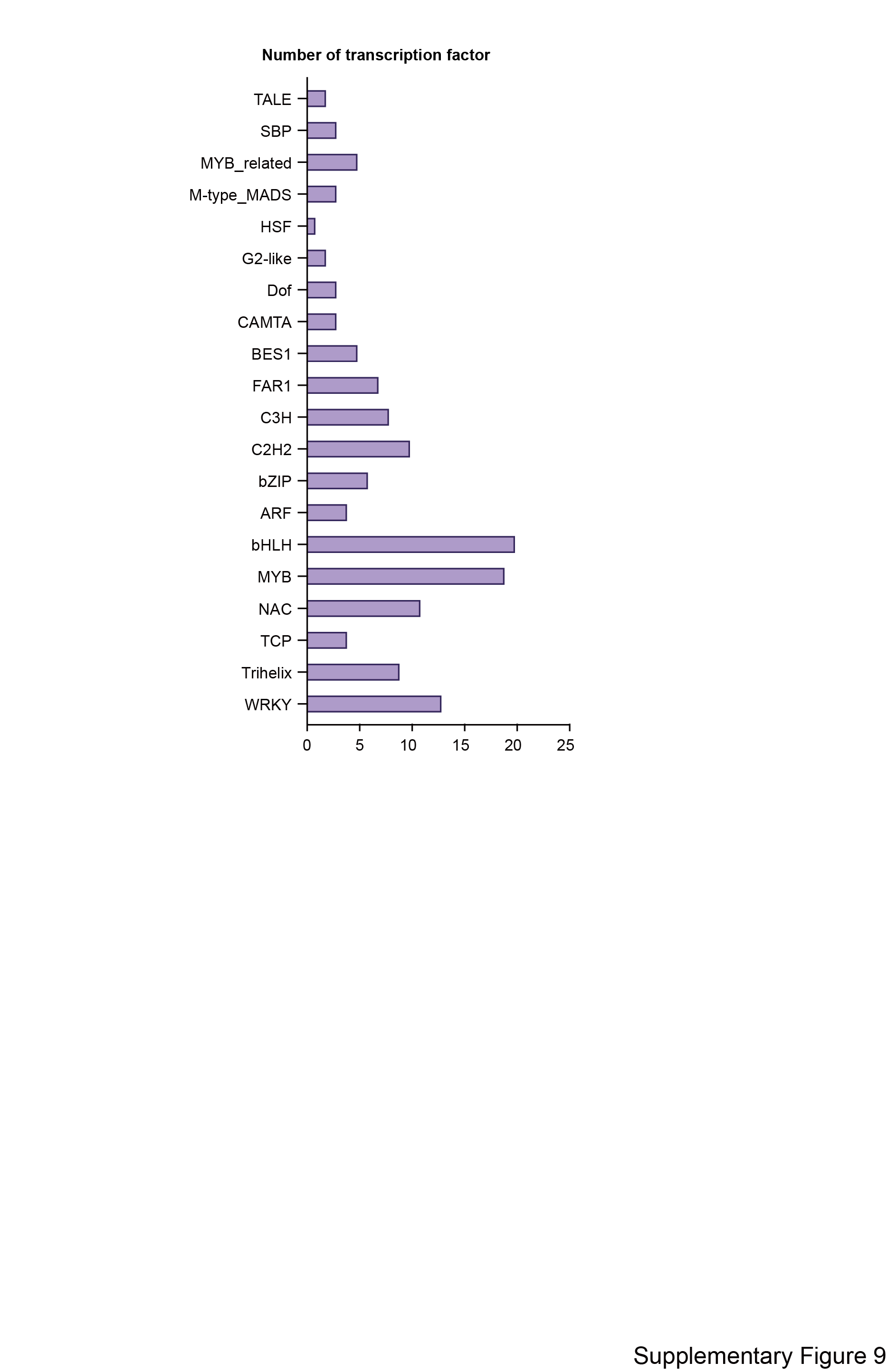

Supplement: Web_Material_uhaf307 [file web_material_uhaf307.zip › Supplementary Figure 9.png]
